# Brain microstructural alterations in the left precuneus mediate the association between KIBRA polymorphism and working memory in healthy adults: a diffusion kurtosis imaging study

**DOI:** 10.1007/s11682-022-00703-z

**Published:** 2022-07-20

**Authors:** Junxia Wang, Sichu Wu, Yi Sun, Jiaming Lu, Jilei Zhang, Yu Fang, Zhao Qing, Xue Liang, Wen Zhang, Qian Chen, Xin Zhang, Bing Zhang

**Affiliations:** 1 Department of Radiology, Nanjing Drum Tower Hospital, The Affiliated Hospital of Nanjing University Medical School, Nanjing, 210008 China; 2 Philips Healthcare, Shanghai, 200040 China; 3 Institute for Brain Sciences, Nanjing University, Nanjing, 210008 China; 4 Department of Radiology, Nanjing Drum Tower Hospital Clinical College of Nanjing Medical University, Nanjing, 210008 China

**Keywords:** Kidney and brain expressed protein, Diffusion kurtosis imaging, Functional magnetic resonance imaging, Amplitude of low-frequency fluctuation, Working memory

## Abstract

Kidney and brain expressed protein (KIBRA) rs17070145 is associated with working memory function and cognitive processes. However, the neural mechanisms underlying these associations are not fully understood. This study aimed to explore the effect of KIBRA polymorphism on brain microstructure and blood oxygenation level dependent (BOLD) fluctuations using diffusion kurtosis imaging (DKI) and resting-state functional magnetic resonance imaging (fMRI) in 163 young adults. We also investigated that whether the imaging alterations mediated the association between KIBRA gene and working memory performance. Voxel-based analysis of DKI data showed that KIBRA C-allele carriers exhibited increased axial diffusivity (AD), radial diffusivity (RD) and mean diffusivity (MD) as well as decreased fractional anisotropy (FA), mean kurtosis (MK) and radial kurtosis (RK) compared with KIBRA TT homozygotes, primarily involving the prefrontal lobe, left precuneus and the left superior parietal white matter. Meanwhile, KIBRA C-allele carriers exhibited decreased amplitude of low-frequency fluctuation (ALFF) in the left precuneus compared to KIBRA TT homozygotes. Mediation analysis revealed that the DKI metrics (MK and RK) of the left precuneus mediated the effect of the KIBRA polymorphism on working memory performance. Moreover, the MK and RK in the left precuneus were positively correlated with ALFF in the same brain region. These findings suggest that abnormal DKI parameters may provide a gene-brain-behavior pathway in which KIBRA rs17070145 affects working memory by modulating brain microstructure in the left precuneus. This illustrates that DKI may provide additional biological information and reveal new insights into the neural mechanisms of the KIBRA polymorphism.

## Introduction

The kidney and brain expressed protein (KIBRA) is highly expressed in the human brain and involved in synaptic plasticity (Schneider et al., 2010), which is essential to human memory. A single nucleotide polymorphism (SNP) (rs17070145, T > C) in the KIBRA gene has been extensively investigated and found to be associated with working memory function and cognitive function(Milnik et al., 2012; Ji et al., 2019; Papassotiropoulos et al., 2006). In a meta-analysis, the significant association of KIBRA with working memory was reported (Milnik et al., 2012). Several neuroimaging studies have shown that particular brain regions were involved in working memory tasks, including the bilateral ventrolateral prefrontal regions and medial temporal lobe (Ranganath, 2010; Cabeza et al., 2002; Braver et al., 2001). Putatively, the effect of KIBRA rs17070145 on working memory performance may be related to such brain regions. However, the KIBRA-related gene-brain-behavior association and its underlying neural mechanisms remain rarely investigated.

The effects of KIBRA rs17070145 on human brain structure and function have been investigated in several neuroimaging studies. KIBRA C-allele carriers exhibited smaller grey matter volume in the medial prefrontal cortex, the anterior cingulate cortex and hippocampus compared with KIBRA TT homozygotes (Witte et al., 2016; Wang et al., 2013; Palombo et al., 2013). In functional magnetic resonance imaging (fMRI) studies, KIBRA C-allele carriers showed increased functional connectivity of the hippocampus(Witte et al., 2016) and increased activation in the hippocampus during memory retrieval tasks(Papassotiropoulos et al., 2006), while *Kauppi* (Muse et al., 2014) and *Muse* (Kauppi et al., 2011) observed increased activation in the hippocampus in KIBRA TT homozygotes. As a reliable indicator of regional spontaneous brain activity, ALFF has been widely used to reflect brain functional activity in several conditions involving impaired cognitive processes, such as Alzheimer's disease (Zheng et al., 2019), amnestic mild cognitive impairment (aMCI) (Zhuang et al., 2019) and leukoaraiosis (LA) (Wang et al., 2019). Nevertheless, little is known about the KIBRA-related effects on the ALFF in cognitively normal adults.

In addition to brain morphology and functional activity study, recent developments in diffusion kurtosis imaging (DKI) have provided a new tool to investigate microstructure in the human brain. Based on the theory of non-Gaussian water molecule diffusion in neural tissues, DKI is more sensitive to subtle alterations of brain microstructure, especially grey matter than conventional diffusion tensor imaging (DTI) (Umesh Rudrapatna, et al., 2014). Both diffusion and kurtosis parameters (e.g., fractional anisotropy (FA), mean kurtosis (MK), radial kurtosis (RK)) can be extracted from DKI data (Jensen & Helpern, 2010). The kurtosis metrics are believed to reflect the heterogeneity of the intravoxel diffusion environment and microstructural complexity. MK is a representative parameter of DKI, which is sensitive and stable enough to reflect subtle changes in brain microstructural complexity. Evidence supported that increased kurtosis indicates increased cellular microstructural density, such as with cytotoxic edema or the growth of tumor cells(Hui et al., 2012). In contrast, decreased kurtosis in degenerative diseases often suggests myelin destruction or cell loss(Struyfs, 2015). DKI is widely used in clinical research, such as patients with Alzheimer's disease (Gong et al., 2014), stroke (Weber et al., 2015) and Parkinson's disease (Marco et al., 2012). We suppose DKI may reveal new insights into the effect of KIBRA polymorphism on the brain microstructure, which is also related to human cognition.

The use of fMRI and DKI techniques may help to further investigate brain microstructure and function involved in memory processing, which could provide an important step towards understanding the pathway between genotypes and memory performance. Our hypothesis was that KIBRA polymorphism modulates brain microstructure and spontaneous brain activity to influence memory function in the healthy adults. The present study explored how the KIBRA rs17070145 polymorphism influences brain microstructure and spontaneous brain activity through a combination of DKI with resting-state fMRI in 163 young adults. To elucidate how the KIBRA polymorphism effects on brain microstructure and function in turn influences cognitive function, mediation analysis was performed to find the biological gene-brain-behavior pathway.

## Materials and methods

### Participants

The current study was approved by the Medical Research Ethics Committee of Nanjing Drum Tower Hospital, and all participants provided written informed consent after a sufficient understanding of the study procedure. The study included 163 healthy, right-handed young Chinese adults (mean age: 22.9 ± 1.8 years; 53 males and 110 females). The enrolled participants had no history of neurological or psychotic disorders, stroke or head injuries, and none of the participants met the criteria for major depression according to the Beck Depression Inventory (BDI).

### Genotyping

We extracted genomic DNA from white blood cells of each subject using a DNA extraction kit (BioTeke, Beijing, China). We genotyped each subject for KIBRA rs17070145 using PCR technology with the support of the BGI Tech Solutions Beijing Liuhe Company. The PCR primer sequences of KIBRA rs17070145 were as follows: forward sequence: 5′ ACGTTGGATGTAAAAATGGTGAGCGCCAGC 3′; reverse sequence: 5′ ACGTTGGATGTGTGGAATCTCTTGACCCAG 3′. The distribution of KIBRA rs17070145 genotypes (7 CC homozygotes; 62 CT heterozygotes; 94 TT homozygotes) in our study was in line with previous studies that the frequency of KIBRA CC homozygotes is lower among Asians than Caucasians (Papassotiropoulos et al., 2006). Therefore, the subject pool in our study was divided into two genotypic subgroups: C-allele carriers (including C/C and C/T) and TT homozygotes.

### Cognitive assessment

An N-back behavioral task was employed to test participants’ working memory function. The task consisted of blocks of two conditions that differed in terms of working memory load (1-back and 3-back) using E-Prime 2.0 software (Psychology Software Tools, Pittsburgh, PA, USA). In each condition, there were 60 letters displayed on the computer screen, and each one was presented for 200 ms followed by an interval time of 1800 ms. Before the experiment began, the subjects had time to practice under the instruction of the experimenter. If the letter on the screen was the same as the one that was presented one (1-back) or three (3-back) letters earlier, participants pressed the right arrow button with their middle finger. Otherwise, they pressed the left arrow button. The participants were asked to respond within 2000 ms. We then collected the data for correct responses in the 1-back and 3-back conditions. In our study, we used the index of accuracy for the analysis and calculated the correct responses for the 3-back minus 1-back (Δ accuracy) condition to assess the working memory capacity of each participant. All cognitive behavioral data were transformed into z values for the statistical analyses.

### MRI data acquisition

MRI data were collected on a 3-Tesla (3 T) MRI scanner using a 32-channel phased-array head coil (Ingenia, Philips Healthcare, Best, The Netherlands). DKI data were collected using a single-shot spin echo echo-planar imaging sequence with the following parameters: repetition/echo time (TR/TE) = 12334/94 ms; matrix = 128 × 128; field of view (FOV) = 256 × 256 mm^2^; in-plane resolution = 2 × 2 mm^2^; slice thickness = 3 mm; no gap, axial slices = 50; 20 encoding diffusion directions with 3 b values (b = 0, 1000 and 2000s/mm^2^) for each direction. Resting-state fMRI images were acquired using single-shot gradient echo echo-planar imaging. The parameters were as follows: TR/TE = 2000/30 ms; FOV = 220 × 220 mm^2^; matrix = 64 × 64; flip angle = 90°; slice thickness = 3 mm; gap = 1 mm; 40 interleaved transverse slices; 180 time-points excluding first 5 dummy scans for T1 equilibrium. Structural images were acquired with high-resolution T1-weighted three-dimensional fast field echo (3D-FFE) sequence (TR/TE = 7.3/3.3 ms; inversion time = 450 ms; FOV = 256 × 256 mm^2^; matrix = 256 × 256; flip angle = 12°; slice thickness = 1 mm; no gap; 188 sagittal slices).

### Imaging processing

#### DKI data preprocessing

We used Diffusional Kurtosis Estimator (DKE version 2.0.0, http://nitrc.org/projects/dke) to process the DKI data and generate parametric maps, including diffusion parametric maps of axial diffusivity (AD), radial diffusivity (RD), mean diffusivity (MD), FA and kurtosis parametric maps of axial kurtosis (AK), MK, RK and kurtosis fractional anisotropy (KFA). After that, we performed a voxel-based analysis (VBA) using FSL (https://fsl.fmrib.ox.ac.uk/fsl/fslwiki) and the detailed steps were as follows: a) remove the non-brain tissue from the b = 0 s/mm^2^ image and T1-weighted image of each subject; b) register the skull-stripped T1-weighted image to the b = 0 s/mm^2^ image for each subject using affine linear transformation; c) normalize the T1-weighted images to the Montreal Neurologic Institute (MNI) space; d) apply the transformation matrices to the corresponding diffusion and kurtosis maps and resampled to 2 mm isotropic resolution; e) smooth all images with a 6-mm full width half maximum (FWHM) Gaussian kernel.

#### Resting-state fMRI data preprocessing

We used the Statistical Parametric Mapping 12 (SPM12) and the Data Processing and Analysis of Brain Imaging (DPABI) toolbox (http://rfmri.org/dpabi) to preprocess the resting-state fMRI data. The detailed steps were as follows: a) discard the first 10 time points of the scan; b) correct for motion; c) co-register with high-resolution anatomical image; d) normalize to the MNI brain template (3 mm × 3 mm × 3 mm); e) smooth with an 8-mm Gaussian smoothing kernel; f) perform temporal bandpass filtering (0.01–0.08 Hz). ALFF was also calculated using DPABI software with the following steps: a) convert the time series of each voxel to the frequency domain using a fast Fourier transform and then obtain the power spectrum; b) compute the square root of each frequency of the power spectrum and then obtain the ALFF value; c) divide the value of ALFF of each voxel by the global mean ALFF value for standardization.

### Statistical analyses

All demographic data and cognitive scores analyses were performed using SPSS software (version 25.0 SPSS, Chicago, IL, United States). Group differences regarding demographic and cognitive data were examined using independent sample t-tests for continuous variables or chi-square tests for categorical variables. All results were reported as the mean ± standard deviation (SD), and the significance level was set as *p* < 0.05.

To investigate intragroup differences of the DKI and ALFF parametric maps, we used voxel-based independent sample t-tests in the DPABI toolbox with age, sex and years of education as nuisance covariates. Multiple comparison correction was performed using Gaussian random field (GRF) correction (DKI: voxel level *p* value < 0.01, cluster level *p* value < 0.01; ALFF: voxel level *p* value < 0.01, cluster level *p* value < 0.05). Then the mean values of brain regions showing significant differences between groups were extracted for further statistical analyses.

We performed the mediation analysis by using the SPSS PROCESS macro to test the associations among KIBRA rs17070145 genotype, DKI maps, ALFF maps and working memory scores (Hayes & Rockwood, 2017). In our study, we defined the KIBRA gene as the independent variable, the mean value of each region of interest (ROI) extracted from DKI and ALFF parameters as the mediators, and the n-back accuracy scores as the dependent variables. Age, sex and years of education were included as covariates. The parameters were as follows: model number = 4, bootstrap sample = 5000, bootstrap confidence interval (CI) method = bias corrected, confidence level for CI = 95%. If the 95% CI did not contain zero, we reached a conclusion that there was a significant mediation effect (*p* < 0.05).

## Results

### Demographic and behavioral data

The demographic and behavioral data of KIBRA rs17070145 genotypic subgroups are summarized in Table 1. The genotype distributions for the KIBRA SNP rs17070145 were in the Hardy–Weinberg equilibrium (χ^2^(2) = 0.76, *p* < 0.05). The grouping method rein the present study was consistent with previous studies involving Chinese young subjects(Li et al., 2020; Wang et al., 2013) and Japanese Alzheimer’s disease (AD) patients(Hayashi et al., 2010). No significant differences were observed in terms of age, sex, years of education, BDI scores and working memory performance (all *p* > 0.05, see Table 1 for details).

**Table 1:** Demographic and behavioral data of 163 participants across KIBRA rs17070145 genotypic groups

	KIBRA rs17070145 genotypic group		T/χ^2^ value	*P* values
	C (*n* = 69)	TT (*n* = 94)		
Sex (M/F)	24/45	29/65	0.280	0.597
Age (years)	22.79 (1.92)	23.00 (1.79)	0.692	0.490
Education (years)	16.31 (1.59)	16.78 (1.66)	1.806	0.073
BDI	4.07 (4.47)	4.22 (4.95)	0.200	0.842
1-back accuracy	0.905 (0.067)	0.919 (0.038)	1.484	0.141
3-back accuracy	0.772 (0.144)	0.764 (0.154)	-0.354	0.724
Δ accuracy	-0.133 (0.149)	-0.155 (0.147)	-0.927	0.355

Data are presented as mean (standard deviation). *KIBRA* kidney and brain expressed protein; *M* male; *F* female; *BDI* Beck Depression Inventory. Δ accuracy, 3-back accuracy minus 1-back accuracy

### Group differences in diffusion and kurtosis metrics derived from DKI data

**Fig. 1:**
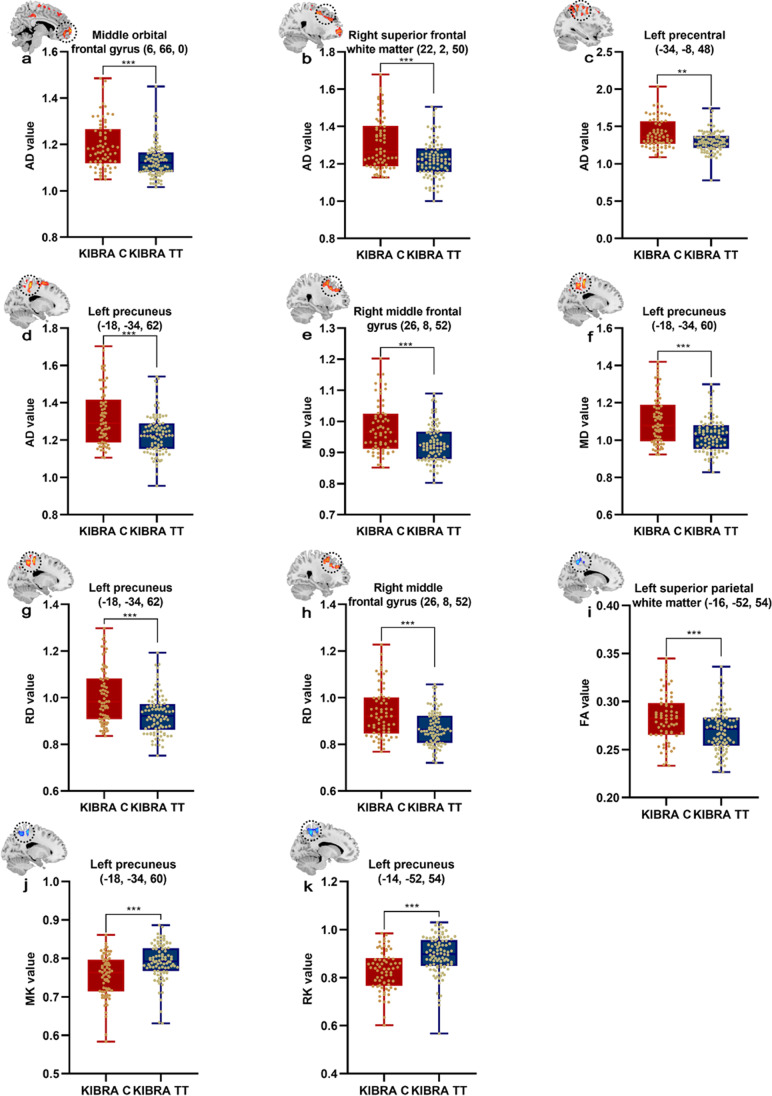
Comparison of diffusion and kurtosis metrics between KIBRA C and TT groups (with Gaussian random field correction, voxel *p* < 0.01, cluster *p* < 0.01). Voxel-based analysis results showed regions with increased AD, MD and RD (the red corresponding brain areas marked by circles) and regions with reduced FA, MK and RK (the blue corresponding brain areas marked by circles) in KIBRA C-allele carriers as compared to KIBRA TT subjects. The MNI coordinates are marked below or beside each significant brain region. Age, sex and years of education were included as covariates. *** *p* < 0.001, ** *p* < 0.01. AD, axial diffusivity; MD, mean diffusivity; RD, radial diffusivity; FA, fractional anisotropy; MK, mean kurtosis; RK, radial kurtosis

Table 2 and Fig. 1 show the brain areas with significant differences in diffusion and kurtosis metrics derived from DKI data between KIBRA C-allele carriers and TT homozygotes with age, sex and years of education as covariates. Compared with KIBRA TT homozygotes, KIBRA C-allele carriers had significantly increased AD, MD and RD in the prefrontal lobe and left precuneus. Meanwhile, FA in the left superior parietal white matter, MK and RK in the left precuneus were lower in the KIBRA C-allele carriers than the KIBRA TT homozygotes (with GRF correction, voxel *p* < 0.01, cluster *p* < 0.01). There were no significant findings in AK and KFA between the two groups (*p* > 0.05).

**Table 2:** Brain regions showing significant intragroup differences in diffusion and kurtosis parameters

DKI metrics	Cluster no	Cluster size/mm^3^	Peak F value	MNI coordinates			Corresponding brain regions
				X	Y	Z	
AD	1	912	4.667	6	66	0	Middle orbital frontal gyrus
AD	2	1645	4.258	22	2	50	Right superior frontal white matter
AD	3	995	4.028	-34	-8	48	Left precentral white matter
AD	4	1739	5.219	-18	-34	62	Left precuneus
MD	1	1387	4.141	26	8	52	Right middle frontal gyrus
MD	2	2119	5.153	-18	-34	60	Left precuneus
RD	1	2402	4.996	-18	-34	62	Left Precuneus
RD	2	862	4.141	26	8	52	Right middle frontal gyrus
FA	1	409	-4.624	-16	-52	54	Left superior parietal white matter
MK	1	729	-4.854	-18	-34	60	Left Precuneus
RK	1	758	-4.702	-14	-52	54	Left Precuneus

*AD* axial diffusivity; *MD* mean diffusivity; *RD* radial diffusivity; *FA* fractional anisotropy; *MK* mean kurtosis; *RK* radial kurtosis; *MNI* Montreal Neurological Institute. Gaussian random field correction, voxel *p* < 0.01, cluster *p* < 0.01

### Group differences in ALFF maps

**Fig. 2:**
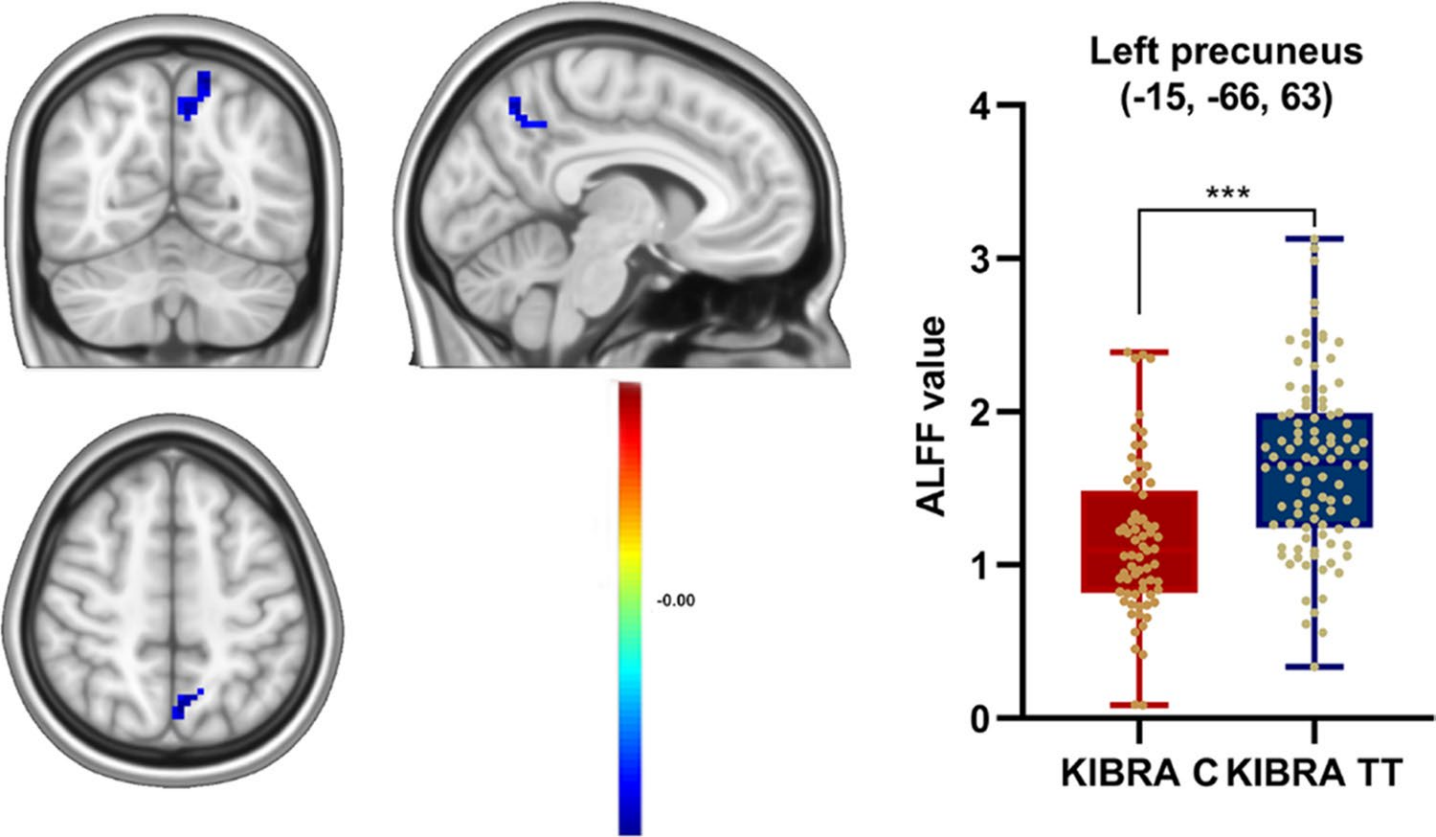
Voxel-based independent sample t-test corrected for age, sex and years of education indicated decreased ALFF in the left precuneus in KIBRA C-allele carriers compared with TT homozygotes (with Gaussian random field correction, voxel *p* < 0.01, cluster *p* < 0.05, x = -15, y = -66, z = 63). *** *p* < 0.001. ALFF, amplitude of low-frequency fluctuation

We found intragroup differences in ALFF maps across KIBRA genetic polymorphisms with age, sex and years of education as covariates. KIBRA C-allele carriers exhibited significantly lower ALFF in the left precuneus than KIBRA TT homozygotes (x = -15, y = -66, z = 63; t = -3.58; cluster size = 92, with GRF correction, voxel *p* < 0.01, cluster *p* < 0.05) (Fig. 2).

### Mediation analysis

**Fig. 3:**
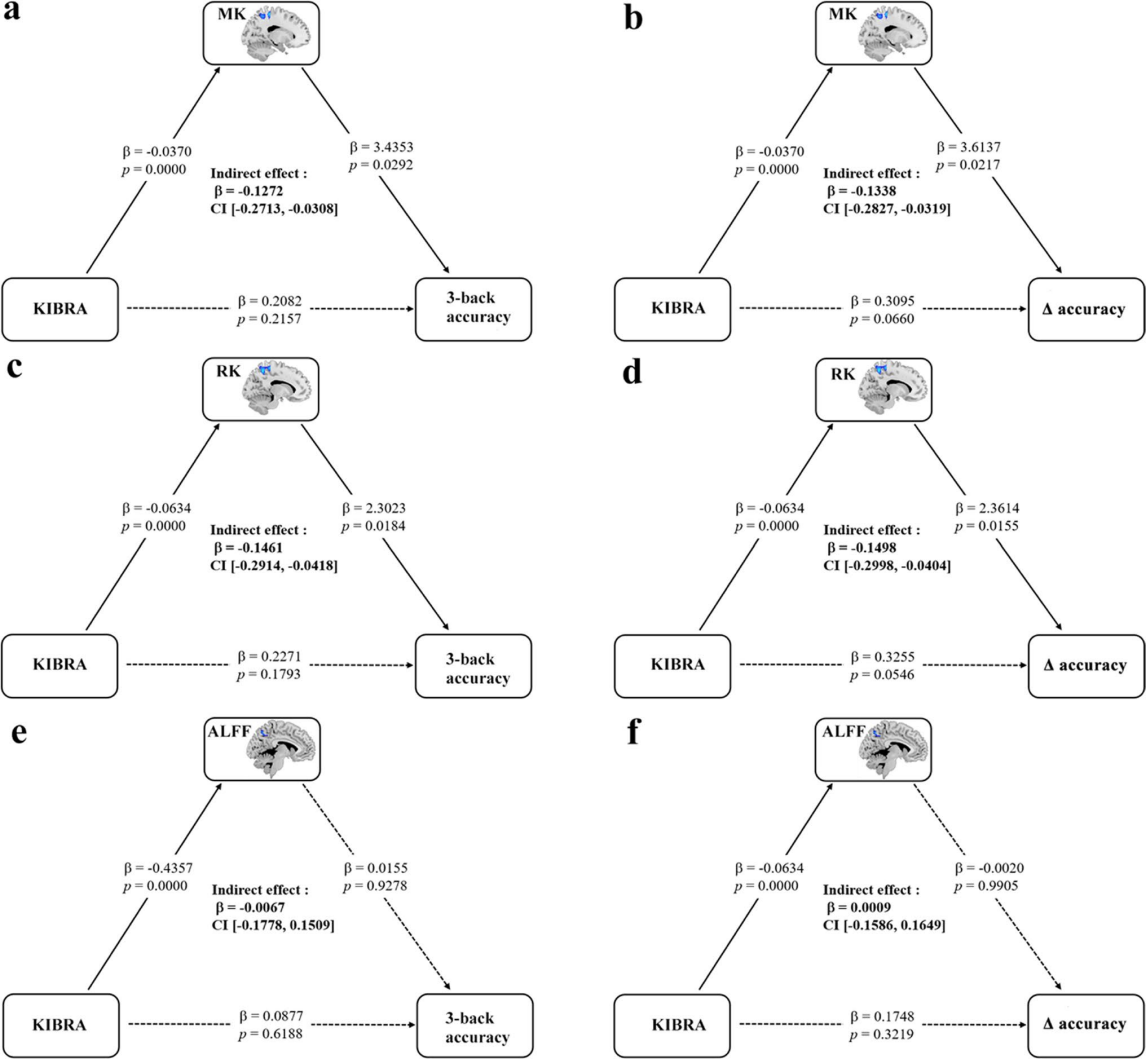
Mediation factors analysis for the associations among KIBRA rs17070145, MK, RK and ALFF in the left precuneus and working memory (3-back accuracy and Δ accuracy (3-back minus 1-back)). The working memory data were transformed to z-scores. Standardized β-coefficients were derived from the mediation models controlling for age, sex and years of education. MK and RK mediated the association between KIBRA rs17070145 and working memory, while ALFF did not exhibit a moderating effect. MK, mean kurtosis; RK, radial kurtosis. ALFF, amplitude of low-frequency fluctuation

**Fig. 4:**
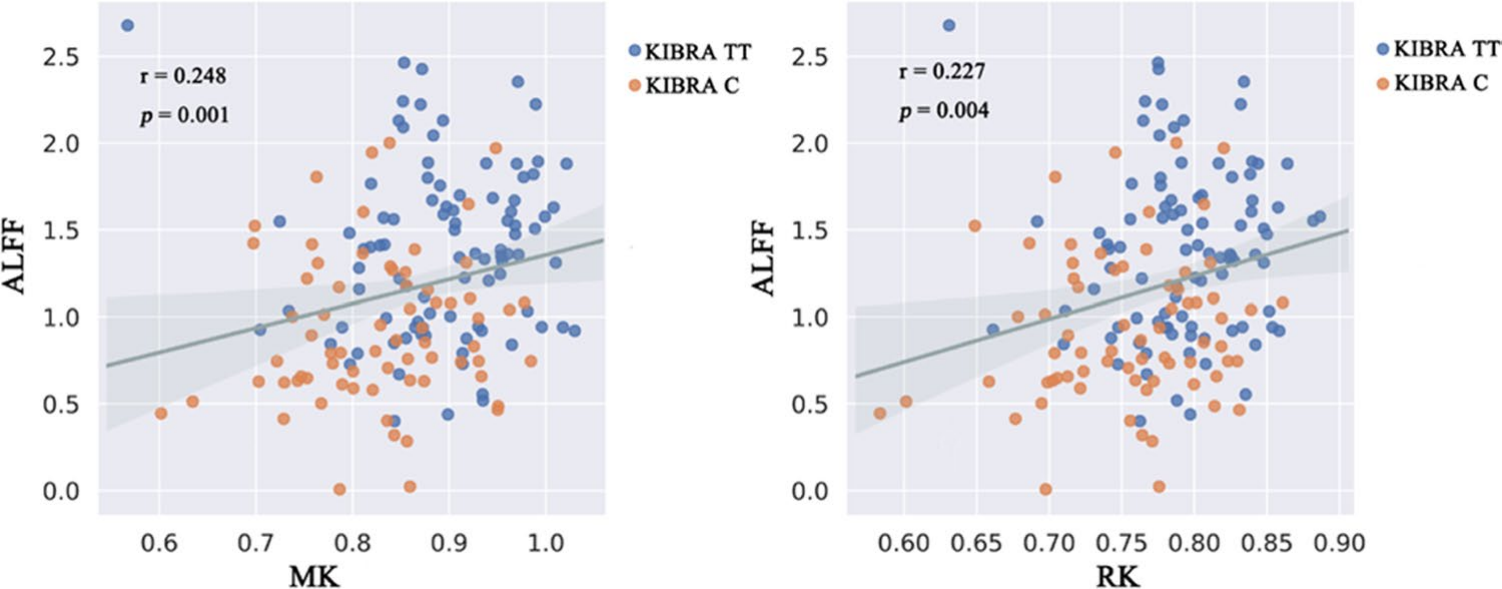
The correlations between DKI maps and ALFF in the left precuneus. The MK, RK in the left precuneus were significantly positively correlated with ALFF (*p* < 0.05)

All ROIs of DKI with intragroup differences were included in the mediation analysis model controlling for age, sex and years of education. Mediation analysis showed a significant indirect effect from KIBRA to working memory mediated by the MK in the left precuneus (3-back accuracy: β = -0.1272, 95% CI [-0.2713, -0.0308]; Δ accuracy (3-back minus 1-back): β = -0.1338, 95% CI [-0.2827, -0.0319]) (Fig. 3) and RK in the left precuneus (3-back accuracy: β = -0.1461, 95% CI [-0.2914, -0.0418]; Δ accuracy (3-back minus 1-back): β = -0.1498, 95% CI [-0.2998, -0.0404]) (Fig. 3). However, we did not find any mediation effect of ALFF in the left precuneus and other diffusion and kurtosis parameters from KIBRA to working memory. Moreover, we found that MK, RK in the left precuneus were significantly positively correlated with ALFF (*p* < 0.05, Fig. 4).

## Discussion

In this study, we found that the KIBRA C-allele carriers exhibited increased AD, RD, MD and decreased FA, MK and RK compared with the KIBRA TT homozygotes in several regions, primarily including the prefrontal lobe, left precuneus and left superior parietal white matter. Meanwhile, the KIBRA C-allele carriers exhibited lower ALFF in the left precuneus than the KIBRA TT homozygotes. Mediation analysis revealed that the DKI metrics (MK and RK) in the left precuneus mediated the association between KIBRA polymorphism and working memory performance, while ALFF in the left precuneus did not exhibit a moderating effect. These findings suggest that the KIBRA polymorphism influences brain microstructure and function in healthy adults. And in some way, DKI metrics may provide additional and meaningful information in the gene-brain-behavior pathway beyond that provided by resting-state fMRI.

There were significant differences between KIBRA C-allele carriers and TT homozygotes in diffusion and kurtosis parameters in several regions as mentioned above. These were key regions associated with memory function(Papassotiropoulos et al., 2006), and some of them were in line with the brain macrostructural changes observed in KIBRA rs17070145 polymorphism, such as the frontal and occipital lobe(Stickel et al., 2017). KIBRA is involved in several cellular functions, particularly synaptogenesis and influences synaptic plasticity as well as human memory. The increased diffusion parameters with decreased FA indicated demyelination and loss of axons in the white matter. The decreased kurtosis parameters reflected the decreased microstructural complexity with the loss of neuron cell bodies, synapses and dendrites in grey matter (Chen et al., 2011; Hu et al., 2017; Schmierer et al., 2007; Sykova, et al., 2005). Therefore, in our study, the increased AD, MD, RD and decreased FA, MK and RK in the memory-related regions may be indicators for the impairments in microstructural integrity and decreased microstructural complexity in KIBRA C-allele carriers. These findings may provide new insights into the neural mechanism of the effect of KIBRA polymorphism on the brain microstructure.

Meanwhile, the KIBRA C-allele carriers exhibited decreased ALFF in the left precuneus compared with the KIBRA TT homozygotes. This is the first study to find KIBRA-related BOLD fluctuation changes in the precuneus, which is a core brain region in the default mode network (DMN), and an important hub in the resting-state brain connectome that has an association with human memory. ALFF is considered to be the spontaneous activity of brain function under the resting state (Zang et al., 2007). In our study, the decreased ALFF in the left precuneus indicated decreased excitability and activity in this region in KIBRA C-allele carriers. This was in line with one positron emission tomography (PET) study carried out in cognitively normal elderly individuals, which found that KIBRA C-allele carriers exhibited lower glucose metabolism than TT homozygotes in the precuneus (Corneveaux et al., 2010). These findings suggested that KIBRA C-allele carriers, despite the cognitively normal individuals, exhibited decreased brain activity in memory-related regions. Thus, it can be seen that the brain microstructure and spontaneous activity in KIBRA C-allele carriers were significantly different from those of TT homozygotes. Although no significant differences were found in the working memory performance between KIBRA C-allele carriers and TT homozygotes, we speculate that healthy individuals may maintain normal cognition through structural or functional compensatory mechanisms in some other brain regions.

The present study showed that the MK and RK in the left precuneus mediated the association between KIBRA rs17070145 and working memory. As a representative parameter of DKI, MK reflects the complexity of the microstructure. Meanwhile, as a radial parameter, RK is more sensitive to the diffusion of water molecules (Marrale et al., 2016). MK and RK are sensitive and stable enough to reflect subtle changes in brain microstructure. In our study, abnormal DKI parameters can provide a gene-brain-behavior pathway in which KIBRA rs17070145 affected working memory by modulating the brain microstructure of the left precuneus. Previous studies have suggested that the precuneus had relationship with working memory (Savoldi et al., 2020; Ren et al., 2019), which were consistent with our findings. Though ALFF in the left precuneus did not mediate the association between KIBRA and working memory, we found MK and RK in the precuneus were positively correlated with ALFF in the same brain region. We speculate that there may be complex physiological mechanisms and functions in the same brain region. Changes in brain microstructure often cause alterations in corresponding functions. However, when there is a slight change in the cell structure, the blood oxygen changes in the gene-brain-behavior pathway are not significant enough to detect. In summary, these findings illustrate that DKI may provide additional biological information and reveal new insights into the neural effects of the KIBRA polymorphism.

There are several limitations in our study. First, the sample size may be somewhat small. In further studies, a larger sample should be collected. Second, we did not control for other memory-related gene polymorphisms, such as APOE and COMT, which may have ignored those genetic interaction effects and bias our results. Third, the subjects recruited in our study were healthy young adults. The same approach will be used in a larger sample of the elderly population in a future study.

## Conclusions

In summary, the present study explored the brain microstructural and functional abnormalities in KIBRA rs17070145 polymorphism in healthy young adults using DKI and resting-state fMRI. KIBRA C-allele carriers exhibited increased AD, RD, MD and decreased FA, MK, RK and ALFF compared with KIBRA TT homozygotes in several memory-related brain regions. Importantly, the MK and RK in the left precuneus mediated the association between KIBRA rs17070145 and working memory. These findings suggest that KIBRA rs17070145 has influence on the brain microstructure and spontaneous activity of brain function. Moreover, brain microstructural alterations are sensitive and reliable for detecting changes in cognitive performance in the KIBRA rs17070145 population.
